# Parental educational level and cardiovascular disease risk factors in schoolchildren in large urban areas of Turkey: Directions for public health policy

**DOI:** 10.1186/1471-2458-5-13

**Published:** 2005-02-04

**Authors:** Bike Kocaoglu, George Moschonis, Maria Dimitriou, Maria Kolotourou, Yasar Keskin, Haydar Sur, Osman Hayran, Yannis Manios

**Affiliations:** 1 Department of Nutrition & Dietetics, Harokopio University of Athens, 70, E. Venizelou Ave,17671 Kallithea, Athens, Greece; 2 Department of Tourism Administration, School of Applied Disciplines, Bogazici University, Istanbul, Turkey; 3 Department of Health Education, University of Marmara School of Health Education, Kartal Devlet Hastanesi Yani, Cevizli, Kartal, Istanbul, Turkey

**Keywords:** Educational level, parents, adolescents, cholesterol, developing country, diet, physical activity

## Abstract

**Background:**

It is widely accepted that the development of atherosclerosis starts at an early age. However, there are very few studies evaluating the prevalence of the common clinical and behavioral cardiovascular disease (CVD) risk factors among children, especially in developing countries. The aim of the present cross-sectional survey was to evaluate the distribution of blood lipid profile and various behavioral (i.e. dietary habits, physical activity status) factors related to CVD risk and its relationships to paternal (PEL) and maternal educational level (MEL) among primary schoolchildren in Turkey.

**Methods:**

In three major metropolises in Turkey (Istanbul, Ankara and Izmir), a random sample of 1044 children aged 12 and 13 years old was examined. ANOVA was applied to evaluate the tested hypothesis, after correcting for multiple comparisons (Tukey correction).

**Results:**

After controlling for energy and fat intake, physical activity status and Body Mass Index (BMI), it was found that mostly PEL had a significant positive effect for most of the subgroups examined (Lower vs. Higher and Medium vs. Higher) on TC and HDL-cholesterol and a negative effect on TC/HDL ratio for both genders. Furthermore, both boys and girls with higher PEL and MEL were found to have higher energy intake derived from fat and protein than their counterparts with Medium and Lower PEL and MEL, while the opposite was observed for the percentage of energy derived from carbohydrates.

**Conclusions:**

Our study provides indications for a possible association between an adverse lipid profile, certain dietary patterns and Higher PEL and MEL among schoolchildren in Turkey. These findings underline the possible role of social status, indicated by the degree of education of both parents, in developing certain health behaviors and health indices among Turkish children and provide some guidance for Public Health Policy.

## Background

Cardiovascular disease (CVD) is the primary cause of mortality in developed countries and generates a major burden of morbidity throughout life [1,2]. Additionally, CVD emergencies have become the leading cause of death in developing countries as well [3,4]. Prospective and retrospective studies have shown that CVD risk factors, namely obesity, the lipid profile, unhealthy diets and sedentary lifestyle, have their roots in childhood and tend to track into adulthood [5-8]. Therefore, early identification and understanding of behavioral and physiological variables related to CVD are essential, so that appropriate interventions can be targeted to children, minimizing the risk of developing the disease later in life.

The continuing modernization and technological advancement of the developing world has brought about rapid lifestyle changes (e.g. fast-food culture, caloric dense diets, sedentary lifestyle), which are known to have a major impact on the development of CVD and other chronic diseases [8,9]. Many investigators have pointed out social and economic status differences, indicated either by the type of school attended (i.e. public vs. private), by the region of residence (i.e. rural vs. urban), or by the level of parental education (primary school vs. University) as important determinants that appear to influence the prevalence of CVD risk factors in both developed and developing world [10-12]. Currently conducted cross-sectional studies have indicated that various socioeconomic factors are inversely associated with CVD morbidity and mortality in developed countries [11,13], while in developing countries, existing data on socioeconomic conditions and the prevalence of CVD are inconsistent [14-16]. The way in which parental educational level seems to exert its effect on health indices and health behaviors differentiates and therefore, should not be considered constant over time and geographic regions [17]. Together health behaviors with social and economic status indices are the outcome of a dynamic and interactive process among several parameters such as education, income, wealth, local culture, beliefs and practices and since none of those parameters stay constant over time and region so do these indices and the way they influence health [18,19]. This observation indicates the need for periodic assessment of these conditions in each population in order to have a clear picture of the social and economic variables and their effects over the populations' health which could effectively guide Public Health Policy in achieving "health for all" and social justice [18].

As far as Turkey is concerned, the available data on the possible effect of parental educational level on the clustering of CVD risk factors among children are limited. Turkey, which has experienced rapid urbanization and industrialization in the past few decades, is a middle-income country, located between Europe and Asia and bordering the Mediterranean, Aegean and Black Seas. The purpose of the present study was to examine the influence of PEL and MEL on CVD risk factors, namely the lipid profile and behavioral indices, among children of Turkey. Expanding our knowledge and understanding on the interrelationship of all these parameters could effectively guide Public Health Policy in early prevention of CVD risk factors in childhood.

## Methods

### Sampling

The first phase of this cross-sectional study took place during March-May 2001 and the second phase during January-May 2002. The study population consisted of primary schoolchildren aged 12 and 13 years old, living in three urban areas of Turkey, namely Istanbul, Ankara and Izmir. Out of totally 1320 6^th ^grade primary school children registered in the selected schools, 1044 pupils (510 from Istanbul, 289 from Ankara, 245 from Izmir, 49.2% males, 50.8% females) were finally examined. Inclusion of subjects was on a voluntary basis; prior to acceptance, children's parents or guardians were fully informed about the objectives and methods of the study and were asked to sign a consent form.

The study population was selected from nine primary schools (three from each city) using the multi-stage sampling method. Schools were selected taking into consideration the available records of the Ministry of National Education and the National Statistical Center of Turkey, in an attempt to obtain a representative sample from the overall population. In the case of Ankara and Izmir data were collected from three public schools respectively, while in the case of Istanbul sample came from one private and two public schools. All children in the same class were invited to participate in the study to avoid ethical problems.

Approval to conduct this survey was granted by the Ethical Committee of Marmara University and the Turkish Ministry of Education.

### Data collection

Baseline data was collected during face-to-face interviews with children by a team of trained personnel. The data collected from the children consisted of physiological indices, namely biochemical and behavioral indices, such as dietary habits, estimation of energy and nutrient intake and physical activity assessment. These data are presented in more detail below.

### Demographic characteristics

A coded questionnaire was developed and administered to the study participants, in order to obtain information on socioeconomic conditions and their personal characteristics, such as family size, parental academic qualifications, occupation and car ownership. Both Paternal (PEL) and Maternal Educational Level (MEL) were classified into three categories. "Lower" PEL and MEL corresponded to illiterate, literate with no formal education and to primary school graduates. "Medium" PEL and MEL was attributed to those parents, who had completed junior high school or high school, whereas the category of "Higher" PEL and MEL included parents who possessed a college or a university diploma.

### Biochemical indices

Early morning venous blood samples were obtained from each child for biochemical screening tests, following a 12-h overnight fast. Professional staff performed venipuncture, using vacutainers to obtain 10 ml of whole blood. Blood was centrifuged for plasma separation at the local University Hospital and 1.5 ml aliquots were pipetted into plastic Eppendorf tubes and stored at -80°C. When blood collection from each city was completed, all samples were sent to Marmara University, Faculty of Health Education, where the actual biochemical analysis took place.

Total cholesterol (TC) was determined using Allain's method [20], while Fossati's method was used for triglycerides (TG) determination [21]. High Density Lipoprotein-cholesterol (HDL-C) was measured by the heparin-manganese precipitation method. Low Density Lipoprotein-cholesterol (LDL-C) was calculated as follows:

LDL-C = TC - (HDL-C + TG/5) [22].

### Anthropometrical measurements

Body weight was measured using a digital scale (Seca) with an accuracy of ± 100 g. Subjects were weighed without shoes, in the minimum clothing possible, i.e. underwear. Standing height was measured without shoes to the nearest 0.5 cm with the use of a commercial stadiometer, with the shoulders in relaxed position and arms hanging freely [23]. Body Mass Index (BMI) was calculated by dividing weight (kg) by height squared (m^2^).

### Dietary assessment

Food consumption of children was assessed by the 24-hour recall technique on three consecutive days. Dietary data was collected from children during a face-to-face interview with a trained dietician. Dieticians were trained as a group to minimize inter-observer variation. During the interview, food models and photos of common Turkish dishes of various portions, as well as household cups and measures were used to define amounts, in order to obtain as accurate information as possible, regarding the type and amount of food and beverages consumed during the previous day. Macronutrient and micronutrient intake were calculated using the food database available in Marmara University, Faculty of Health Education. This database contains Turkish food composition tables for all food, including cooked Turkish dishes. Information on processed foods was obtained from food companies and national as well as international fast food chains.

### Physical activity assessment

Physical activity during school hours and leisure time was assessed using a standardized physical activity diary completed by the children for two consecutive weekdays and one weekend day. A member of the research team crosschecked diary information during daily interviews. The diary was constructed for searching various physical activities ranging from mild to vigorous [24], while the time spent for each type of activity was recorded in hours.

Activities were classified into two groups, namely Sedentary and Light Activities (< 4 METs) and Moderate to Vigorous Physical Activities (MVPA) (> 4 METs). Typical activities in the Sedentary and Light category were watching TV, board and computer games, studying and extra curricular classes (e.g. music, language), etc. The MVPA category included activities such as walking, bicycling, rhythmic-gymnastics, dancing, basketball, soccer, athletics, tennis, swimming, running up and down, jumping rope and general participation in active outdoors games. Given the age group, MVPA was defined as continuous vigorous activity causing sweating and heavy breathing for periods longer than 15 minutes, but with occasional breaks in intensity, rather than the strict aerobic definition of 20 continuous minutes appropriate for adults.

### Statistical analysis

Descriptive statistics of continuous variables were expressed as the Mean ± standard deviation (s.d.), as well as median and 25^th ^and 75^th ^percentile for not normally distributed variables. The 2- sample Z test was used to compare proportions regarding values above normal range among the different categories of parental education for both genders. One- way analysis of variance (ANOVA) with a Tukey post- hoc analysis and multivariate analysis of variance (MANOVA) controlling for certain confounding factors, was conducted to determine whether differences derived from the comparison of continuous variables among the educational categories were statistically significant. For not normally distributed variables the non- parametric univariate Mann-Whitney test was used to verify the statistical significance of the existing differences among the three educational categories. All analyses were conducted using the SPSS 10.0 statistical software package for Windows. In all analyses a 5% significance level was used.

## Results

Table 1 summarizes the mean serum lipid values determined for the study participants by gender and parental educational level, as well as the significant differences observed among the three educational level categories. According to these findings Higher PEL and MEL boys and girls were found to have significantly higher mean levels of TC and HDL-C, but lower mean TC to HDL-C ratio, compared to their Medium and Lower PEL and MEL peers. However, after adjusting for sex, energy intake, fat intake, BMI and MVPA some of the differences presented in Table 1 lost their significance (data not shown). More specifically, when PEL was used as a grouping variable HDL-C remained higher for Higher PEL boys and girls, compared to those of Medium PEL (p = 0.010 for boys and p < 0.001 for girls) as well as for Higher PEL girls compared to those of Lower PEL (p < 0.001). A similar observation applied for TC to HDL-C ratio, which remained lower for Lower PEL boys and girls, compared to Medium (p = 0.021 for boys and girls) and to Lower PEL ones (p = 0.022 for boys and p = 0.005 for girls). When mean serum lipid levels where compared on the basis of MEL categories, most of the differences observed in Table 1 lost their significance, since only HDL-C and LDL-C remained higher for Higher MEL boys, compared to Medium (p = 0.018) and to Lower MEL ones (p = 0.020) respectively.

**Table 1:** Biochemical indices by gender and parental educational level

**Father's educational level (PEL)**	**BOYS**			
		
**Serum Lipids**	**Lower (n = 174)**	**Medium (n = 264)**	**Higher (n = 95)**	**p-value (ANOVA)**
TC (mg/dl)	159.7 ± 30.2	167.6 ± 31.6^a^	169.3 ± 30.9^b^	0.007
HDL-C* (mg/dl)	53.8 ± 31.9	54.2 ± 14.7	60.3 ± 15.2^b,c^	<0.001
LDL-C cholesterol (mg/dl)	90.3 ± 27.2	95.6 ± 30.0	96.2 ± 29.7	0.930
Triglycerides* (mg/dl)]	90.6 ± 49.0	85.3 ± 38.0	85.4 ± 43.4	0.568
TC/ HDL-C ratio	3.23 ± 0.85	3.28 ± 0.98	2.96 ± 0.86^b,c^	0.011

**Mother's educational level (MEL)**				
**Serum Lipids**	**Lower (n = 276)**	**Medium (n = 209)**	**Higher (n = 46)**	**p-value (ANOVA)**

TC (mg/dl)	162.3 ± 32.1	168.9 ± 29.4^a^	168.8 ± 34.0	0.036
HDL-C* (mg/dl)	54.0 ± 15.9	53.7 ± 13.5	60.0 ± 18.0^b,c^	0.033
LDL-C cholesterol (mg/dl)	90.9 ± 28.6	97.2 ± 29.1^a^	99.6 ± 31.8	0.017
Triglycerides* (mg/dl)]	88.0 ± 45.2	87.2 ± 42.4	85.0 ± 39.1	0.851
TC/ HDL-C ratio	3.16 ± 0.83	3.34 ± 1.01	3.02 ± 0.97	0.023

**Father's educational level (PEL)**	**GIRLS**			
		
Serum Lipids	**Lower (n = 164)**	**Medium (n = 281)**	**Higher (n = 66)**	p-value (ANOVA)

TC (mg/dl)	168.9 ± 29.8	172.0 ± 34.2	175.5 ± 30.5	0.308
HDL-C* (mg/dl)	51.1 ± 11.5	52.9 ± 12.5	61.5 ± 20.0^b,c^	<0.001
LDL-C cholesterol (mg/dl)	103.2 ± 85.4	99.9 ± 32.8	95.5 ± 31.9	0.609
Triglycerides* (mg/dl)]	95.3 ± 42.3	95.5 ± 49.6	98.1 ± 45.9	0.879
TC/ HDL-C ratio	3.44 ± 0.84	3.40 ± 0.92	3.06 ± 0.89^b,c^	0.008

**Mother's educational level (MEL)**				
**Serum Lipids**	**Lower (n = 259)**	**Medium (n = 227)**	**Higher (n = 27)**	**p-value (ANOVA)**

TC (mg/dl)	168.4 ± 32.2	174.2 ± 32.7	179.7 ± 28.1^b^	0.046
HDL-C* (mg/dl)	51.6 ± 13.9	54.9 ± 12.8^a^	56.2 ± 13.7^b^	0.002
LDL-C cholesterol (mg/dl)	100.6 ± 72.0	99.8 ± 33.0	107.4 ± 26.3	0.799
Triglycerides* (mg/dl)]	95.2 ± 42.1	97.4 ± 51.9	81.5 ± 35.1	0.194
TC/ HDL-C ratio	3.41 ± 0.85	3.32 ± 0.93	3.38 ± 0.95	0.488

^a ^p < 0.05 Medium SES vs. Low SES

^b ^p < 0.05 High SES vs. Low SES

^c ^p < 0.05 High SES vs. Medium SES

* Parameter was log transformed.

Table 2 presents the proportions of children with serum lipid levels above the cut-off points, proposed by the NCEP [25]. According to these findings the prevalence of children found to be in "borderline or high risk" according to their TC and LDL-C serum levels, was significantly higher in Higher PEL and MEL boys and girls, compared to their Medium and Lower PEL and MEL peers, respectively. Furthermore, the prevalence of children with HDL-C levels below 35 mg/dl was found to be lower in Higher PEL boys, compared to those of Medium and Lower PEL.

**Table 2:** Percentage of children characterized as "borderline or high risk" by gender and parental educational level.

**Father's educational level (PEL)**	**BOYS**			**GIRLS**		
	
**Borderline or High Risk**	**Lower % (n = 174)**	**Medium % (n = 264)**	**Higher % (n = 95)**	**Lower % (n = 164)**	**Medium % (n = 281)**	**Higher % (n = 66)**
TC ≥ 170	32.0	43.6^a^	49.5^b^	45.4	50.2	60.6^b^
LDL-C ≥ 110	21.4	28.0	25.3	33.2	37.7	30.3
HDL-C<35	7.8	5.7	0.0^b,c^	6.1	4.5	3.0
TC/ HDL-C>4.5	9.7	9.1	5.3	10.7	10.9	9.1

**Mother's educational level (MEL)**						
**Borderline or High Risk**	**Lower % (n = 276)**	**Medium % (n = 209)**	**Higher % (n = 46)**	**Lower % (n = 259)**	**Medium % (n = 227)**	**Higher % (n = 27)**

TC ≥ 170	36.2	45.7^a^	50.0	45.1	54.8^a^	70.4^b^
LDL-C ≥ 110	21.2	29.8^a^	32.6	32.5	38.0	40.7
HDL-C<35	5.8	6.5	0.0	6.8	4.9	5.7
TC/HDL-C>4.5	7.4	11.0	8.7	8.5	12.2	14.8

* Lipids cut-off points in mg/ dl (according to NCEP)

^a ^p < 0.05 Medium SES vs. Low SES

^b ^p < 0.05 High SES vs. Low SES

^c ^p < 0.05 High SES vs. Medium SES

Dietary and physical activity characteristics of the under study population are reported in Tables 3 and 4. Both boys and girls with Higher PEL were found to have higher intakes of energy derived from fat and protein than Lower and Medium PEL boys (p = 0.002 and p = 0.021) and girls (p = 0.007 and p = 0.010) respectively. Regarding boys of Higher MEL, they were found to have lower total energy intake (p = 0.005) but higher percentages of energy derived from fat (p = 0.002) and protein (p = 0.001), compared to boys of Lower and Medium MEL. The percentage of energy derived from fat was also found to be higher for Medium and Higher MEL girls, compared to those of Lower MEL (p = 0.001). On the other hand the contribution of carbohydrates to the total daily energy intake was found to be higher for Lower MEL boys (p = 0.025) and girls (p = 0.001), compared to Medium and Higher MEL children. With the exception of MVPA, which was found to be higher only for Medium MEL boys, compared to Lower MEL ones (P < 0.05), no other statistically significant differences were observed.

**Table 3:** Energy and macronutrient intakes by gender and parental educational level.

**Father's educational level (PEL)**	**BOYS**			
		
**Dietary Indices**	**Lower (n = 171)**	**Medium (n = 266)**	**Higher (n = 99)**	**p-value (ANOVA)**
Energy Intake* (Kcal/ day)	2031.4 ± 556.1	2017.7 ± 603.5	1952.3 ± 642.9	0.373
Fat Intake (%)	29.7 ± 7.2	30.2 ± 6.3	32.4 ± 5.5^b,c^	0.002
Protein Intake (%)	13.3 ± 2.3	13.3 ± 2.6	14.1 ± 2.5^b,c^	0.021
CHO Intake (%)	40.0 ± 10.9	39.3 ± 10.5	39.9 ± 9.3	0.739

**Mother's educational level (MEL)**				
**Dietary Indices**	**Lower (n = 274)**	**Medium (n = 213)**	**Higher (n = 46)**	**p-value (ANOVA)**

Energy Intake* (Kcal/ day)	2000.7 ± 546.9	2059.6 ± 650.8	1754.8 ± 557.5^b,c^	0.005
Fat Intake (%)	29.7 ± 6.6	30.6 ± 6.4	33.2 ± 6.9^b,c^	0.002
Protein Intake (%)	13.3 ± 2.4	13.4 ± 2.6	14.8 ± 2.8^b,c^	0.001
CHO Intake (%)	41.1 ± 8.6	40.6 ± 10.7	38.4 ± 10.2^b^	0.025

**Father's educational level (PEL)**	**GIRLS**			
		
**Dietary Indices**	**Lower (n = 157)**	**Medium (n = 278)**	**Higher (n = 73)**	**p-value (ANOVA)**

Energy Intake* (Kcal/ day)	1913.3 ± 562.9	1926.8 ± 585.8	1796,0 ± 413,0	0.414
Fat Intake (%)	30.5 ± 6.7	31.0 ± 6.3	33,2 ± 6,4^b,c^	0.007
Protein Intake (%)	12.9 ± 2.6	12.9 ± 2.5	13,8 ± 2,2^b,c^	0.010
CHO Intake (%)	38.4 ± 11.5	36.7 ± 10.4	36,6 ± 7,9	0.189

**Mother's educational level (MEL)**				
**Dietary Indices**	**Lower (n = 260)**	**Medium (n = 220)**	**Higher (n = 31)**	**p-value (ANOVA)**

Energy Intake* (Kcal/ day)	1889,3 ± 554,7	1915.5 ± 533.4	1780.2 ± 609.5	0.404
Fat Intake (%)	30,2 ± 6,5	31.9 ± 6.5^a^	33.4 ± 6.5^b^	0.001
Protein Intake (%)	12,8 ± 2,4	13.0 ± 2.6	13.6 ± 2.3	0.260
CHO Intake (%)	38,8 ± 11,2	35.7 ± 9.6^a^	35.4 ± 10.0	0.001

^a ^p < 0.05 Medium SES vs. Low SES,

^b ^p < 0.05 High SES vs. Low SES,

^c ^p < 0.05 High SES vs. Medium SES

* Parameter was log transformed

**Table 4:** Physical activity by gender and parental educational level.

**Father's educational level (PEL)**		**BOYS**			**GIRLS**		
		
**Physical Activity**		**Lower (n = 174)**	**Medium (n = 267)**	**Higher (n = 94)**	**Lower (n = 157)**	**Medium (n = 281)**	**Higher (n = 71)**
MVPA (hours/week)	Median (25th – 75th percentile)	6.7 (2.3–11.1)	7.0 (3.5–11.6)	7.0 (3.2–11.6)	2.3 (0.0–7.0)	3.5 (0.0–7.0)	1.2 (0.0–7.0)
	Mean ± SD	7.3 ± 6.4	7.9 ± 6.1	7.6 ± 6.4	4.1 ± 4.4	4.2 ± 4.5	4.0 ± 5.3

**Mother's educational level (MEL)**							
**Physical Activity**		**Lower (n = 273)**	**Medium (n = 214)**	**Higher (n = 45)**	**Lower (n = 260)**	**Medium (n = 222)**	**Higher (n = 30)**

MVPA (hours/week)	Median (25th – 75th percentile)	5.8 (2.3–10.5)	8.1^a ^(3.5–11.6)	5.8 (2.3–8.1)	2.3 (0.0–7.0)	3.5 (0.0–7.0)	0.6 (0.0–5.2)
	Mean ± SD	7.2 ± 6.4	8.2 ± 6.1^a^	6.7 ± 5.8	3.8 ± 4.3	4.4 ± 4.8	3.5 ± 5.0

^a ^p < 0.05 Medium SES vs. Low SES (Mann Whitney test).

## Discussion

Despite the traditional notions of CVD as a Western disease of "affluence", more than three quarters of global CVD mortality now occurs in middle- and lower- income nations (WHO, 2001) [26]. Furthermore, progression of CVD as well as the risk factors and health behaviors leading to this medical condition seem to be related with various social and economic indices for both adults and children [11,27]. However, prevention seems to be the most effective way to treat CVD and since the roots of the disease are located in childhood, early detection and management of the related risk factors should begin at that age [27,28].

According to the findings of the current study there was a higher prevalence of "borderline or high risk", with respect to TC levels, for children with Medium and Higher parental educational level (Table 2). Several other investigators, who have conducted analogous epidemiological surveys in other developing nations, such as in Philippines, Costa Rica and Iran [29-31], have confirmed these alarmingly high prevalence rates among children living under the supervision and care of better educated parents. Furthermore, in contrast with Western populations where TC levels are often inversely associated with income and education [32,33], but in consistency with several other studies conducted in recent years in Turkey the findings of the current one indicated a positive effect of parental education on serum TC levels [34,35]. The same trend applied for HDL-C levels since, in consistency to the findings of Mahley et al. (2001) [34] for upper socioeconomic status prepubescent subjects, the present study showed that children with Medium and Higher PEL and MEL were found to have higher serum levels of this lipoprotein than those with Lower PEL and MEL.

The effect of diet on serum lipids and lipoproteins has been extensively studied in various clinical trials and epidemiological studies, and although dietary content is clearly an important determinant, several environmental and metabolic factors intervene to modulate the dietary effect (36). Previously conducted surveys in urban regions of Turkey have provided strong evidence that children coming from more affluent and/or well-educated families consumed diets higher in animal protein and fat, while the typical diet of less privileged children was rich in carbohydrates, including cereal grains and sugar (37). These findings, regarding the macronutrient content of the consumed diet, are in consistency with the current study, since children from Medium and Higher PEL and MEL were found to consume higher percentage of energy derived from fat and protein, while the opposite was observed for carbohydrate intake (Table 3). Based on the existing literature (38) we could safely speculate that higher intake of fat, especially saturated, and of protein, originating mostly from animal products, could explain the more unfavorable lipidemic profile, with respect to TC and LDL-C serum levels, among children with Higher PEL and MEL. Additionally the lower HDL-C levels and the higher TC to HDL-C ratio reported for children of Lower PEL and MEL could probably be attributed to the higher ratio of carbohydrates constituting their diet. Indeed the percentage of energy derived from carbohydrates has been inversely associated with HDL-C levels according to the results from several other studies [39,34].

Regarding physical activity, it is well established that higher levels of physical activity are associated with higher HDL-C and lower TC to HDL-C ratio values in both children and adolescents [40-42]. However the current study, in agreement to similar findings of Mahley et al (2001) [34], has not revealed any significant differences with respect to MVPA among children of different PEL and MEL. Still, of great interest are the differences observed in physical activity levels between genders. The present study in consistency with many other studies conducted in the developed world [43-45] revealed that boys were found to devote more time on MVPA than girls. This gender differentiation in physical activity levels should not be attributed to physiological differences between the two sexes but to social and cultural beliefs of parents and teachers as to the types of activities appropriate for boys and girls. Family and society have been shown to influence the level and type of physical activity, girls are engaged in and therefore may determine the lifetime habits with respect to habitual physical activity [45].

The findings of the current study are in agreement with previous surveys conducted in Turkey indicating that parental education, either as a single estimate of social status or combined with other social and/or economic indices, is a "reliable index" of socioeconomic status [34,46]. Both indices (PEL and MEL) utilized in the present study provided similar findings regarding the differences on children's serum lipid levels. These could probably be attributed to dietary differences, since no significant differences were reported for physical activity. Furthermore, higher percentage of energy derived from fat and protein recorded for Higher PEL and MEL children, compared to those with Lower PEL and MEL, in conjunction with the opposite finding regarding carbohydrates, is in agreement with previous studies indicating "limited" access to food by children of lower socioeconomic status [46]. These findings may provide some indication that less privileged children are not only under a greater risk of undernourishment but furthermore, by having a higher ratio of TC to HDL-C, that they combine, at the same time, a high prevalence of a health risk index of affluence. However, due to its cross sectional design the present study could not establish causal-effect relationships, but only generate hypothesis about the possible role of parental education on certain CVD risk factors. Another limitation that has to be considered is the fact that the study's focus was on certain biochemical and behavioral indices, since it has not included other important CVD risk factors such as fasting glucose, insulin, blood pressure and smoking habits.

The underlying influence of socio-economic status on health status is far more complex than diet and physical activity alone, since many other factors interact in producing the differences observed for serum lipids or other health indices. Based on the data provided by the current study it could be concluded that the differences regarding the lipidemic profile of children with different parental educational level could possibly be attributed to the differences observed on their dietary and macronutrient intake. Any attempt for the development and implementation of a health and education programme should consider these findings and set priorities accordingly. However further research is needed in order to develop an effective index for assessing socioeconomic level, as well as in better understanding its multidimensional role on public health. All these will help the public health authorities to develop effective strategies, which will efficiently tackle these health issues early in life.

## Pre-publication history

The pre-publication history for this paper can be accessed here:


